# Full range tuning of the composition of Au/Ag binary nanoparticles by spark discharge generation

**DOI:** 10.1038/s41598-021-84392-6

**Published:** 2021-03-04

**Authors:** Attila Kohut, Lajos Péter Villy, Albert Kéri, Ádám Bélteki, Dániel Megyeri, Béla Hopp, Gábor Galbács, Zsolt Geretovszky

**Affiliations:** 1 Department of Optics and Quantum Electronics, University of Szeged, Dóm sq. 9, Szeged, 6720 Hungary; 2 Department of Materials Science, Interdisciplinary Excellence Centre, University of Szeged, Dugonics sq. 13, Szeged, 6720 Hungary; 3 Department of Inorganic and Analytical Chemistry, University of Szeged, Dóm sq. 7, Szeged, 6720 Hungary

**Keywords:** Design, synthesis and processing, Nanoparticles, Metals and alloys, Synthesis and processing

## Abstract

Gold/silver bimetallic nanoparticles still attract extensive interest due to their favorable properties e.g. in plasmonics or catalysis. We present here a facile and robust way for the production of clean Au/Ag binary nanoparticles (BNPs) with a total control over the composition via the spark discharge nanoparticle generation technique. With the application of pure Ag and Au electrodes, a tuning range of 55 to 90% Au content was achieved, but this can be further extended to the full 0 to 100% range by using a couple of alloyed electrodes. An added benefit of the approach is that either the concentration or the mean particle size can be kept constant at every composition by adjusting the generator parameters. Based on the systematic experimental data collected, a semi-empirical model for the prediction of the Au/Ag BNP composition was also developed. This model was used to calculate the theoretically achievable Au/Ag composition at a given spark parameter set in the parameter range most commonly used in the literature.

## Introduction

Gold and silver nanoparticles (NPs) serve as the workhorses of plasmonic research for decades due to their strong plasmon resonance, which can be excited by visible light^1^. Due to their broad field of applications covering e.g. photothermal therapy^2^, localized surface plasmon resonance (LSPR) based sensing^3–5^, or surface enhanced Raman spectroscopy^5–8^, tremendous work has been done to control their properties and hence their performance. Gold and silver NPs have been synthesized with various shapes including spheres, rods, cubes, boxes, triangles or stars with sizes ranging from a few nanometers to tens of nanometers^9–11^. Mixing these two metals opens up further perspectives in their LSPR-related applications. Au/Ag alloy NPs have a plasmon resonance band between the plasmon bands of pure gold and silver, the position of which can be continuously varied by controlling the alloy composition^12^. In addition to this tunability, Au/Ag alloys effectively combine the high activity of silver with the excellent chemical stability of gold^13^. Au/Ag alloy NPs have found successful applications in e.g. the field of surface enhanced Raman spectroscopy^13,14^, biomedical imaging^12^ or catalysis^15,16^.

The literature describes many possibilities for the synthesis of Au/Ag alloy NPs via either chemical or physical methods. Among all the synthesis routes, co-reduction of Au and Ag precursors is probably the most popular and straightforward^17^. In order to have more control over the reproducibility and size distribution of the NPs, seed-mediated synthesis is often used where the nucleation and growth stages of particle formation are separated^12^. Additional ways to produce Au/Ag alloy NPs via chemical synthesis can be carried out e.g. via microwave heating in a polymer solution^18^, ultrasonic treatment^19^, or photochemical synthesis^20^. Physical methods offer relatively simple alternatives to chemical synthesis, while avoiding the use of reagents, solvents or various precursors. These are typically based on the ablation of a bulk alloy via a laser^21^ or spark discharge^22^. However, the preparation of a bulk target with a predefined composition is often impractical when the tuning of the particle composition is desired.

In the present paper we report on the gas phase synthesis of Au/Ag alloy NPs in various compositions via spark discharge generation, an inherently clean and environmentally friendly method. We demonstrate and investigate the potential of spark discharge nanoparticle generators (SDGs) for the continuous tuning of the composition of Au/Ag alloy NPs, by simply changing the resistance of the discharge circuit. The achievable composition range as well as the underlying physical processes are discussed. The compiled experimental data and theoretical considerations are used to construct a semiempirical mixing model, which can be used to predict the average composition of the Au/Ag BNPs generated in an SDG.

## Results and discussion

### The concept of BNP generation in SDGs from dissimilar elemental electrodes

**Figure 1:**
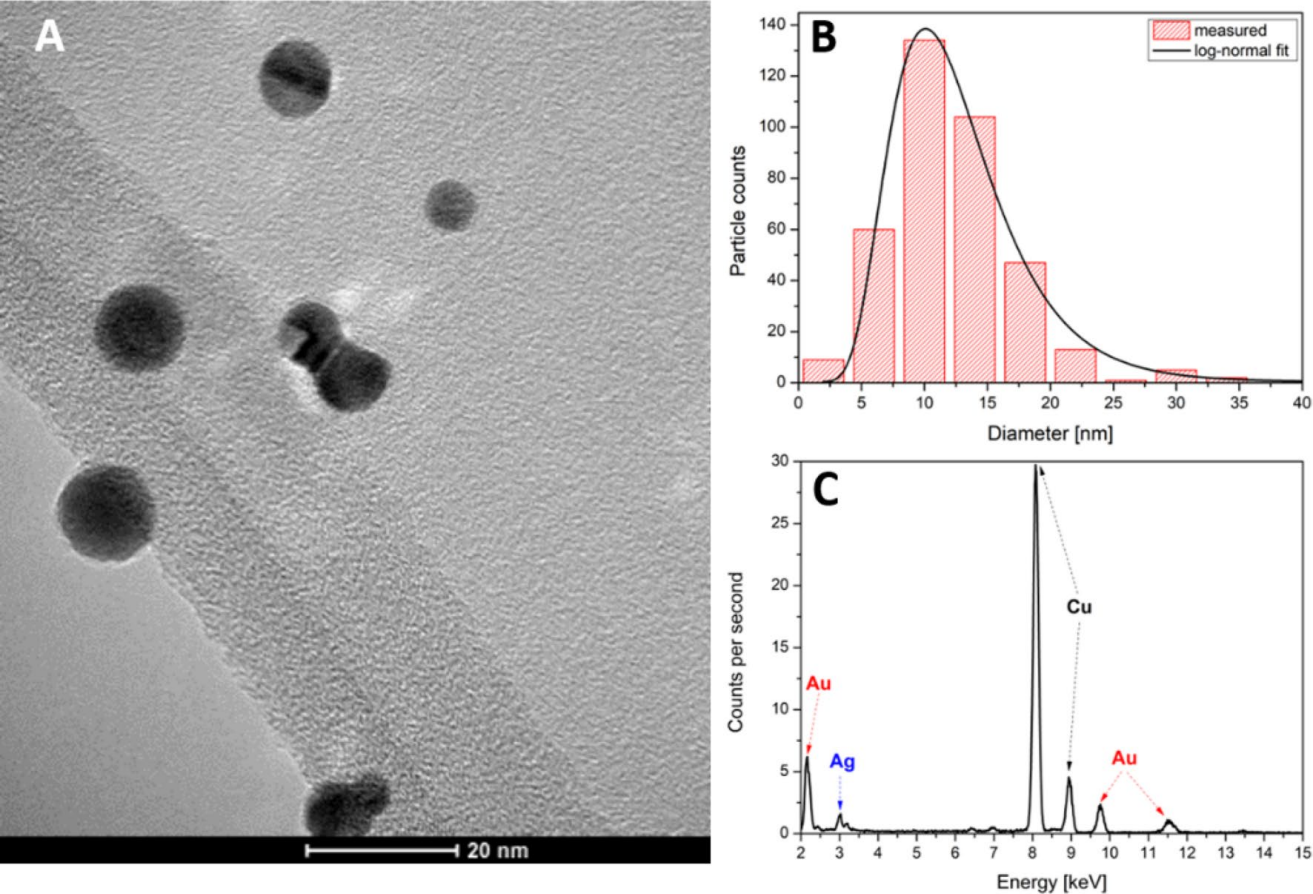
Morphology (**A**), size distribution (**B**), and EDX spectrum (**C**) of Au/Ag BNPs generated in the SDG (1 Ω total resistance, initially anodic Au electrode, 900 °C compaction furnace).

SDGs are based on the initiation of an energetic spark discharge between two electrodes, which are placed in a controlled, flowing—usually inert—gas^23^. As a result of the accompanying high current, the electrode material is released into the gas phase where, after nucleation and growth, NPs are formed^24^. If the electrodes are dissimilar, particles with complex composition can be obtained. The particles formed in the process are proven to contain material from both electrodes, as a consequence of the erosion of both electrodes^25,26^, which stems in the oscillatory nature of spark discharges^27^. The current waveform of a spark follows an exponentially dampening sinusoidal function. This corresponds to the fact that the discharge circuit of an SDG is essentially a serial RLC circuit, where the current is described by the well-known Eq. (1).

1 $$ I\left( t \right) = \frac{{V_{0} }}{{\frac{2\alpha }{{\omega_{0} C}}}} \cdot \frac{\alpha }{{\sqrt {1 - \alpha^{2} } }} \cdot e^{{ - \omega_{0} \alpha t}} \cdot 2 \cdot {\text{sin}}\left[ {\omega_{0} t\sqrt {1 - \alpha^{2} } } \right], $$

where the *V*_*o*_ is the initial voltage at the capacitor in Volts, *α* is the damping factor, defined as $\alpha  = \frac{R}{2} \cdot \sqrt {\frac{C}{L}} ,$ and *ω*_*0*_ is the angular frequency in units of radians per second, defined as ${\omega }_{0}=\frac{1}{\sqrt{LC}}$, where *C* is the capacitance of the capacitor in Farads, *R* and *L* are the total resistance and the inductance of the circuit in Ohm and Henry, respectively. And *t* is the time in s. The polarity of the electrodes (direction of current) changes in every half cycle. Since the material erosion caused by a spark discharge is mainly attributed to the arc stage of the discharge^28^, which is the high-current conducting phase of the spark, polarity reversals result in an alternating erosion of the electrodes. As a result, atoms from both electrodes will be released into the gas phase and will serve as the source for the formation of nanoparticles. It is well known that different electrode materials have different erosion rates during sparking^29,30^; this will also affect the composition of the generated BNPs. Typical morphology of the particles formed in our generator is shown in Fig. 1A. The particles have well-defined spherical shape, due to the heat-induced compaction of the aerosol carried out by the tube furnace (c.f. Fig. 7). As it is also shown in Fig. 1B, the size distribution of the particles follows a lognormal function with a geometric mean diameter of about 11 nm. EDX spectrum of the particles (Fig. 1C) confirm the presence of both gold and silver which, together with the morphology, indicates alloy formation as expected^22^. Please note that the other peaks in the EDX spectrum are originating from the TEM grid used for sampling and the vacuum chamber. Samples shown in Fig. 1 have an average gold content of ca. 77%, which qualitatively reflects the significantly higher erosion rate of gold than silver, already reported^30^. It should be noted that the composition of the SDG-generated BNPs exhibit a certain distribution, meaning that the composition of an individual particle may differ from the average composition, which is originated from the peculiarities of the spark plasma-based process^31^.

### Composition tuning via discharge circuit modification

**Figure 2:**
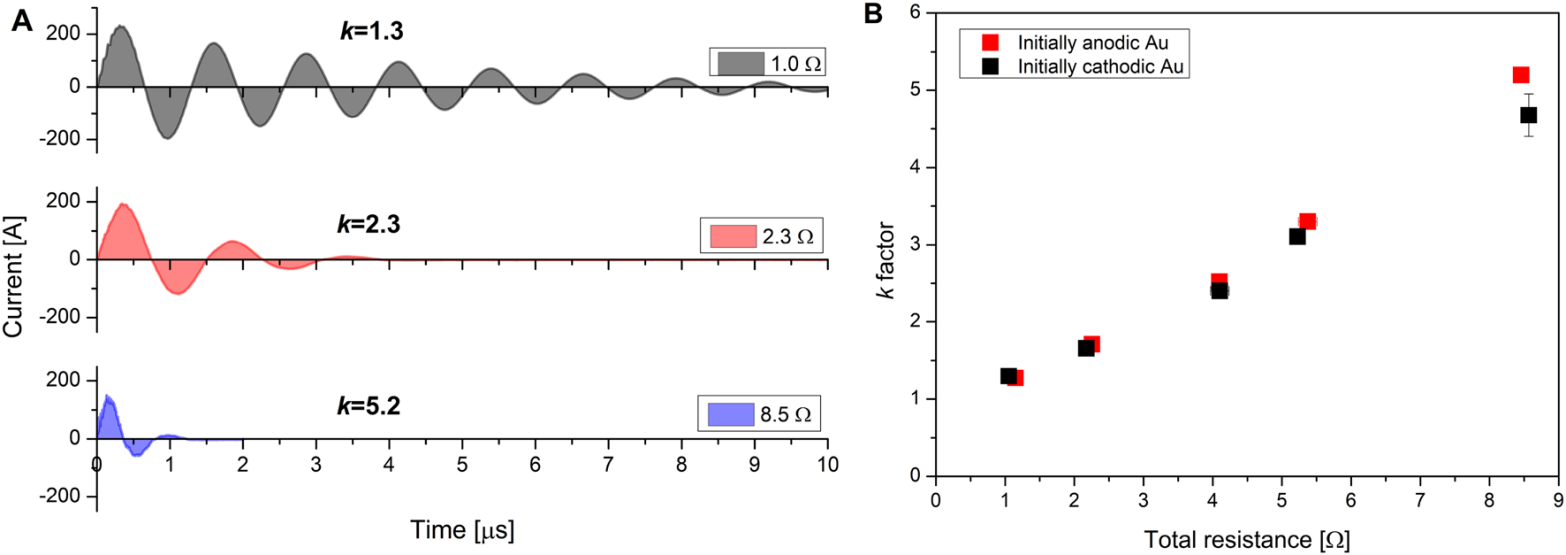
Illustrative current waveforms of the spark discharge measured at different total resistances (**A**). Asymmetry of the current as a function of the total resistance (**B**). See the text for the definition of the asymmetry *k* factor.

It was mentioned in the previous subchapter that the composition of BNPs generated via sparking is affected by the erodibility of the two electrode materials. Nevertheless, this is not the only factor that influences the particle composition. In most cases, the erosion of the initially cathodic electrode is reportedly larger than that of the anodic electrode, even in case of identical chemical composition^30^, which can be taken as the manifestation of that material removal mainly takes place at the cathode. This in turn means that the relative erosion rate of different electrodes will also depend on their initial polarity^32^, which leads to the conclusion that the composition of the generated BNPs can be altered by simply switching the polarity of the electrodes. This, however, only allows for a certain constant shift in the composition. For example, under experimental parameters corresponding to Fig. 2, a swap of the electrodes (making gold the cathode at the start of discharge) leads to a further increase of gold content to ca. 81%. Based on the above, the relative erosion of the two electrodes is linked to their cathodic periods, which can be described by the symmetry of the current waveform (c.f. Fig. 2)^31,32^. It means that by altering the symmetry of the current waveform, the relative erosion of the electrodes and hence the composition of the BNPs can be tuned.

The current waveform can be altered by modifying the resistance, inductance, and/or capacitance of the discharge circuit. Experimentally, the most accessible parameter is resistance. By looking at Eq. (1) it is apparent that the total resistance of the circuit affects the damping factor and thereby the peak current of the circuit, therefore by adding an extra resistor to the serial circuit, the current symmetry and duration of oscillations (number of half-cycles) can be varied.

The effect of increasing circuit resistance on the current waveform is exemplified by Fig. 2A. The top (black) waveform corresponds to the lowest total resistance achievable in our SDG. This is composed of the resistance of the wires, connectors, electrodes, contact points, and the spark plasma itself^33^. When the total resistance is increased, the peak current decreases and the dampening increases, resulting in a lower number of current reversals and a shorter duration of the sparking. It can also be seen that along with an increasing damping, the asymmetry also becomes more pronounced, which is related to the energy delivered to the electrodes in subsequent half-cycles, proportional to the current. For the description of “the degree of asymmetry” and hence the ratio of energy delivered to each electrode in their cathodic periods, Feng et al*.*^31^ introduced the *k* factor:

2 $$k=\frac{\int {I}_{positive}^{2}(t)dt}{\int {I}_{negative}^{2}(t)dt},$$

where *I*_*positive*_ is the current measured in the half-cycles when it has a positive value and *I*_*negative*_ when the polarity is reversed, given that the current probe is installed in a way that the current is positive in the first half-period. As is shown in Fig. 2B, the *k* factor increases along with the increase of the circuit resistance. This means that by changing the resistance of the circuit, a continuous tuning of the current waveform can be achieved.

**Figure 3:**
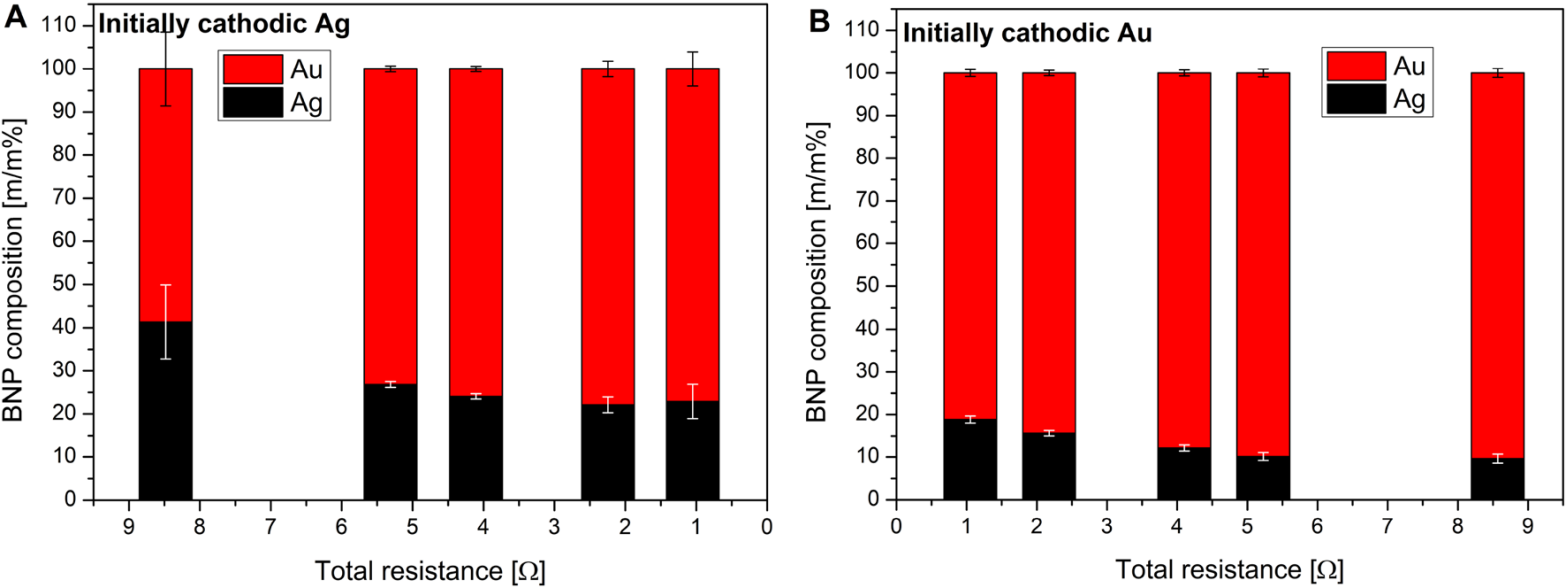
Composition of the generated Au/Ag BNPs as a function of the total circuit resistance when the initially cathodic electrode is silver (**A**) or gold (**B**).

In order to investigate the effect of the variation of the *k* factor on the composition, Au/Ag nanoparticles were generated with the systematic increase of the circuit resistance, for both electrode polarities. Results are shown in Fig. 3. It is apparent that the Au content of the generated nanoparticles is always higher than 50 m/m%, in agreement with the much higher erodibility of the former, as it was mentioned above. It can also be seen in Fig. 3 that the share of the metal corresponding to the initially cathodic electrode polarity increases with the increase of the total resistance, which is in a qualitative agreement with the variation of the *k* factor (cf. Fig. 2B). As evidenced by Fig. 3, combining the polarity change of the electrodes with the modification of the circuit resistance, a total tuning range of 55 to 90 m/m% can be achieved in terms of the Au content.

It should also be mentioned however, that changing the total resistance not only changes the composition of the BNPs, but also their size distribution. We have recently shown that increasing the circuit resistance results in a decrease of nanoparticle size and number concentration, which is a consequence of the energy dissipated on the added resistor and hence the decrease of the energy efficiency of the process^33^. For the present material system and conditions, this is evidenced by data shown in Supplementary Fig. S1A. It is clear that an increase in the resistance shifts the mode of the distribution curve towards smaller diameters, ranging from ca. 18 nm to about 11 nm in the resistance range studied here. For the sake of proper tuning of the composition, this particle size effect needs to be corrected by the adjustment of the spark repetition rate (SRR) and the carrier gas flow rate. As it is demonstrated by the experimental data shown in Supplementary Fig. S1B, the particle size can be kept constant indeed, within ca. 1 nm, by slightly increasing both the SRR and the carrier gas flow rate. It should also be noted that the variation of the SRR does not affect the composition of the binary nanoparticles and could only cause the particle concentration to increase, which has no drawbacks in practice. On the other hand, the above results also indicate that one can keep the particle concentration (yield) constant for the chosen composition by adjusting the SRR and the carrier gas flow rate. As a result of this, the mean particle size will shift, but this may not be inhibiting BNP fabrication for certain applications.

Due to the degrading energy efficiency, it is not practical to increase the resistance above a certain level. Similarly, the resistance cannot be decreased beyond the minimum resistance of the discharge circuit. These two constraints set the practical limits of the tuning range achievable with the present method—in the case of using elemental gold and silver electrodes. However, it has been confirmed that the composition of multielement (alloy or sintered) electrodes is maintained by the generated BNPs during sparking^34,35^, which can be exploited to further expand the Au/Ag composition range by replacing one of the elemental electrodes with an alloy of the two metals. For example, by using a 50:50 m/m% Au/Ag alloy electrode against a silver electrode and using minimum circuit resistance, we could decrease the Au content of the BNPs generated to about 33%. This means that by combining a few electrodes of discrete compositions and introducing additional resistors to the circuit the composition of the generated Au/Ag BNPs can be virtually continuously tuned to any value between 0 and 100 m/m%.

### Development of a spark mixing model

**Figure 4:**
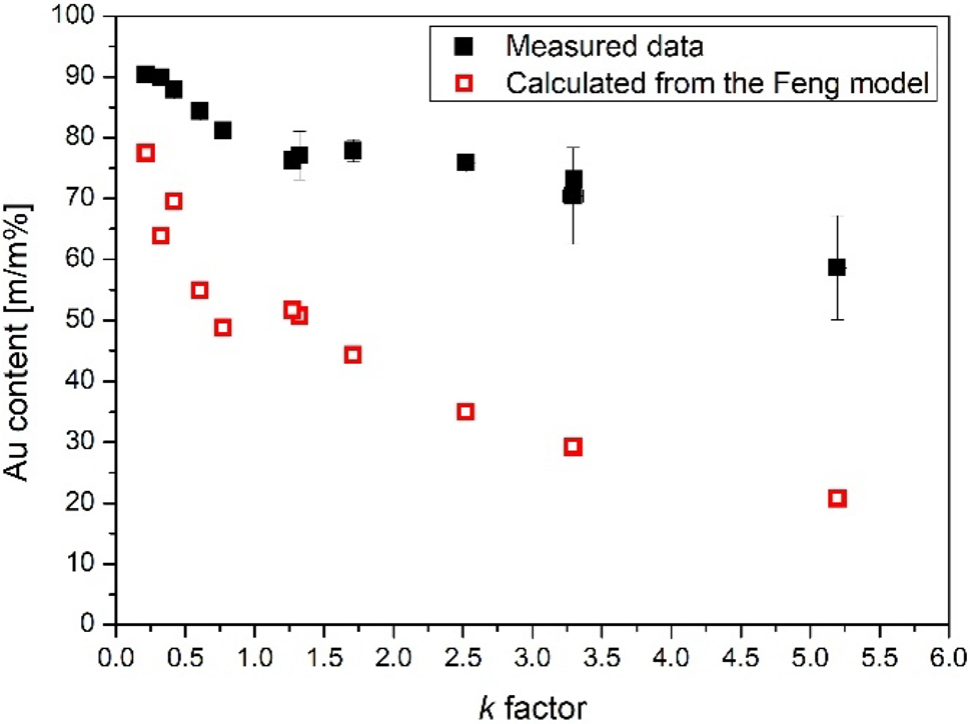
Comparison of the experimentally determined and the predicted, according to Ref.^31^, Au content of the generated BNPs as a function of the *k* factor.

Results shown in Fig. 3 qualitatively support the hypothesis that the mixing ratio between gold and silver is determined by the asymmetry of the current waveform, describable by the *k* factor. For the prediction of the composition of BNPs generated in SDGs, Feng et al*.* proposed the following semi-empirical formula^31^:

3 $$ \varphi_{C} = \frac{1}{{1 + \frac{{C_{A} }}{{k \cdot C_{C} }}}}, $$

where ${\varphi }_{C}$ is the relative mass percent of the initially cathodic electrode, *C*_*A*_ and *C*_*C*_ are calculated from thermal constants of the initially anodic and cathodic electrode, respectively^31^. The Feng model is based on two main assumptions: *i)* the eroded mass is linearly proportional to the energy delivered to each electrodes, described by the integral of the square of the current, and *ii)* only the electrode which is momentarily the cathode is eroded^31^. In Fig. 4 we provide a comparison of these predicted composition values to our experimentally measured data. Please note that the reversal of the initial polarity of the electrodes can be treated formally by taking the reciprocal of the *k* factor calculated for one of the two cases. Here we set *k* to be greater than 1 when Ag is initially cathodic, which brings it to between 0 and 1 when Au is initially cathodic. It can be seen that although the model provides a reasonable prediction for the trend as a function of the *k* factor, the calculated values are far off from the measured values. The relative deviation from the experimentally determined compositions is in the range of 10–65% and the model consistently underestimates the gold content. This could mean that either the *C*_*A*_ and *C*_*C*_ constants fail to properly describe the “erodibility” of the electrode materials, or the energy input is not describable solely by the integral of the square of the current for every pair of electrode materials. A physical explanation for the latter case can be given based on the so called sheath layers of a gas discharge plasma existing in the vicinity of the electrodes^36^. Energy exchange between the plasma and the electrodes is mostly governed by these regions which responsible for the voltage drop over the discharge^37–39^. The energy delivered to the electrodes can be calculated by integrating the product of the voltage drop and the current^39,40^. It should be noted that formally a resistance can be assigned to the sheet layer and hence the energy can be calculated from the integral of the square of the current as in the Feng model. Nevertheless, the voltage has much more physical relevance and therefore literature values can be usually found for the voltage drop. Literature data show that the exact values for voltage drop over a discharge gap depends on the electrode material and the ambient gas as well. Values in the range of about 14–50 V can be found for various metals, which are independent from the current over a relatively broad current range^39–42^. If we consider first that only the momentary cathode is eroded in an SDG—in accordance with the Feng model—the typical cathode fall voltages should be compared for Au and Ag in the present case. The most recent value we found for silver is about 14 V^38^, but unfortunately we are unaware of any relevant measurements in case of gold electrodes. Nonetheless, if we assume that cathode fall voltages are different for gold and silver, the different energy input and hence different erosion rates would explain the strong deviation of the calculated composition values from the measured ones (c.f. Fig. 4).

Up to this point, the contribution of the momentary anode to the total material erosion was neglected. Following the above reasoning about the voltage drop over the interelectrode gap, this assumption is rather nontrivial. Even though the total voltage over the electrode gap is dominated by the cathode fall voltage, the voltage drop in the anode fall region is not negligible either. Hemmi et al. determined the ratio of the cathode and anode fall voltages to be about 3.1 for an arc discharge operated in air between silver electrodes^38^. Moreover, as for the cathode fall, anode fall voltage also found to be dependent on the electrode material and experimental conditions^43^. In a search for experimental evidence on anodic erosion in the present case, the mass of each component (i.e. Au and Ag) eroded can be also looked at. Our ICP-MS analytical data obtained for the average composition of the BNPs allows for a more accurate evaluation of the eroded masses than simple gravimetric measurements typically used in SDG research. Based on this, we are able to compare the mass of Au and Ag converted to aerosol (that is in the portion of aerosol collected on the filter) when either the Au or the Ag electrode is initially cathodic. Assuming that the current is reasonably reproducible at a given total resistance—which is a condition generally fulfilled—the particle mass ratio generated from the initially cathodic and anodic electrodes (denoted as *m*_*cathode*_*/m*_*anode*_) can be obtained from the two measurement sets shown in Fig. 3. As followed from the exclusive cathodic erosion assumption of the Feng model, *m*_*cathode*_*/m*_*anode*_ should be equal to *k* for a given electrode material, according to Eq. (3). For the variation of *m*_*cathode*_*/m*_*anode*_ as a function of *k* both for Au and Ag please see Fig. S2 in the Supplementary Information. It is apparent that while the values for Au reasonably follow the theoretically predicted trend, there is a strong deviation in the case of Ag. This suggests—at least for silver—that a significant anodic erosion is also present. Taking into account the above considerations regarding the potentially different energy input for gold and silver and the presence of anodic erosion in case of silver, we propose the extension of the Feng model to cover these phenomena. To this end, *U*_*-*_ and *U*_+_ are introduced to describe the cathode and anode fall voltages, respectively, and the energy delivered to a given electrode is calculated from the product of the voltage and the integral of the current in the corresponding periods. For example, material removal from an initially cathodic electrode is driven by cathodic erosion in its momentary cathodic periods—i.e. the dissipated energy is calculated by the product of the integral of the oscillatory current when it has positive values and *U*_*-*_ for the given material—and by anodic erosion in its momentary anodic periods, related to the product of the integral of the “negative” current and *U*_+_. For the detailed formalism and equations please see the Supplementary Information. If we consider the data shown in Supplementary Fig. S2 representing the erosion of identical Au or Ag electrodes, one can derive the ratio of cathode and anode fall voltage for a given electrode material:

4 $$ U_{ - / + }^{Ag} = \frac{{m_{C/A} *k^{\prime} - 1}}{{k^{\prime} - m_{C/A} }}, $$

where *m*_*C/A*_ is the *m*_*cathode*_*/m*_*anode*_ ratio and *k*^*’*^ is an asymmetry factor similar to *k*, but it is calculated from the integral of the current, instead of its square (c.f. Eq. (2)). For Ag, Eq. (4) results in a value of 2.7 ± 0.6 with a confidence level of 90%. This agrees reasonably well with the 3.1 value obtained by Hemmi et al*.* for the same electrode material^38^. This also means that in the present case, the anodic erosion of the Ag electrode is about 30% of the cathodic erosion, which is indeed not negligible. As for Au, the average relative deviation between *k’* and *m*_*C/A*_—excluding the highest resistance case—is only ca. 6%, which means that cathodic erosion alone describes the measured masses fairly well, as suggested by Fig. S2. From the equations detailed in the Supplementary Information the relative mass percent of the initially cathodic electrode—i.e. the composition—is the following:

5 $$ \varphi_{C} = \frac{1}{{\frac{1}{{\frac{{C_{A} }}{{C_{C} }} \cdot \frac{{U_{ - }^{C} }}{{U_{ - }^{A} }} \cdot \frac{{k^{\prime} + U_{ + / - }^{C} }}{{U_{ + / - }^{A} \cdot k^{\prime} + 1}}}} + 1}}, $$

where ${U}_{-}^{C}$ and ${U}_{-}^{A}$ are the cathode fall voltage of the initially cathodic and anodic electrode, respectively, and ${U}_{+/-}^{C}$ and ${U}_{+/-}^{A}$ are the ratio of the anode and cathode fall voltages for the initial cathode and anode, respectively. Please note that if we neglect the anodic erosion—i.e. ${U}_{+/-}^{C}={U}_{+/-}^{A}=0$—and the difference between the cathode fall voltages of different electrode materials—i.e. $\frac{{U}_{-}^{C}}{{U}_{-}^{A}}=1$—Eq. (5) takes the same form as Eq. (3). From the above results, we can take that ${U}_{+/-}=1/2.7$ for silver and ${U}_{+/-}=0$ for gold, however we do not have exact values of *U*_*-*_ for gold. Therefore, we adjust the value of $\frac{{U}_{-}^{Au}}{{U}_{-}^{Ag}}$, i.e. the ratio of the cathode fall voltages when the initially cathodic electrode is gold and the initial anode is silver, in order to obtain a good fit of the values calculated from Eq. (5) to the measured compositions. As a cross check, we use the obtained cathode fall voltage ratio to calculate the compositions in the reversed polarity case.

**Figure 5:**
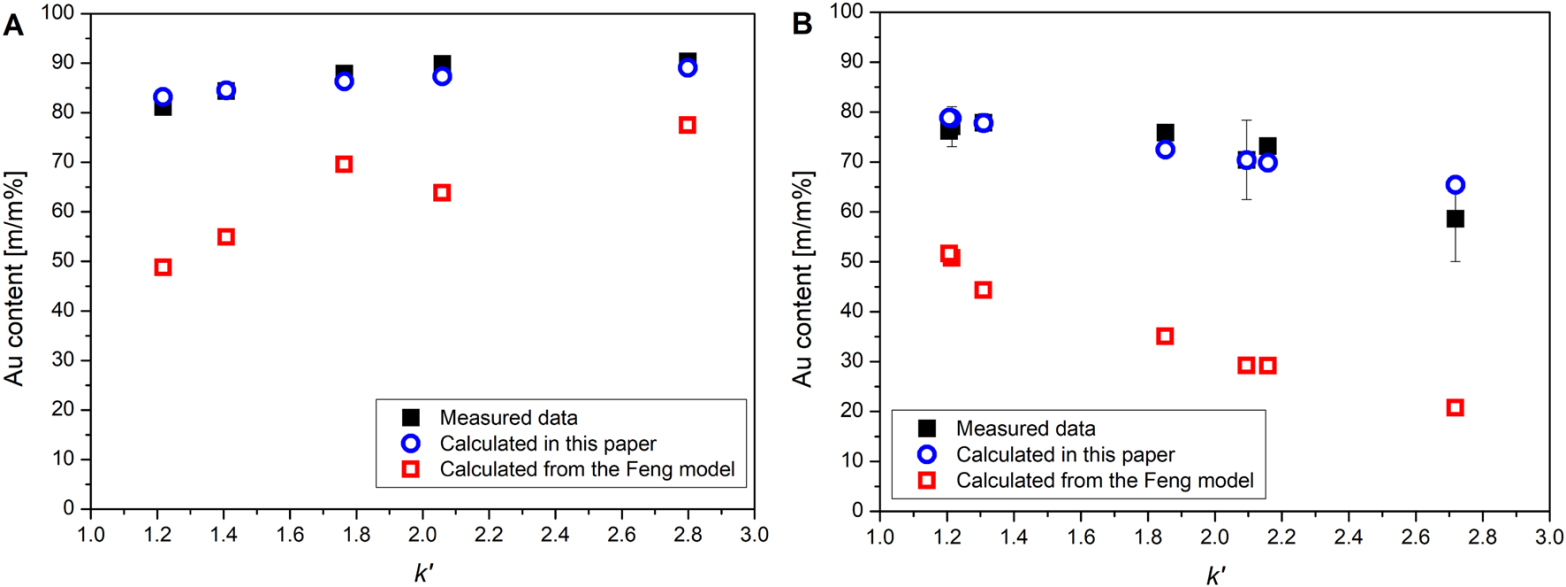
Comparison of the experimentally determined and the predicted, according to Eq. (5) and Ref.^31^, Au content of the generated BNPs as a function of the *k’* factor when initially the cathode is gold (**A**) and silver (**B**).

**Figure 6:**
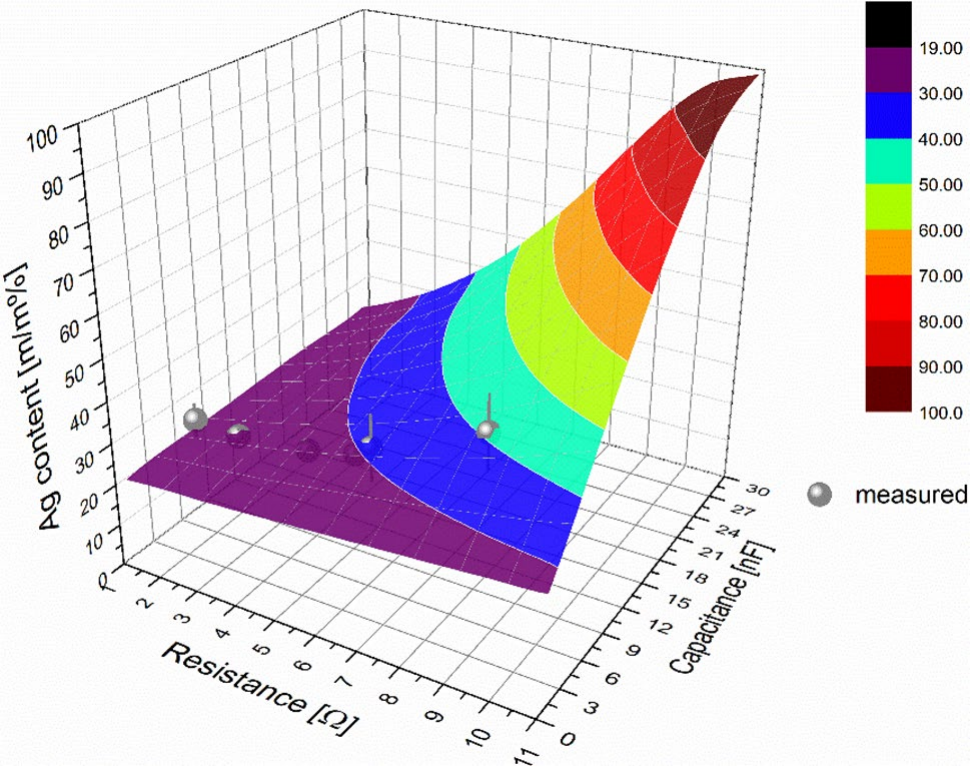
Theoretical Ag content of Au/Ag BNPs achievable in the SDG by varying the total resistance and capacitance of the discharge circuit (calculated from Eqs. (1) and (5). The gold electrode is initially anodic, the total inductance is 1 µH).

The best fit of the measured data for initially cathodic gold electrode was obtained when the $\frac{{U}_{-}^{Au}}{{U}_{-}^{Ag}}$ ratio was set to ca. 4.3, as shown in Fig. 5A. This means that the reciprocal of this value, namely 0.23, should be used to obtain the composition data when the polarity is reversed, i.e. when the initially cathodic electrode is silver. As can be seen in Fig. 5B, the calculated values fit the measured data neatly, strongly indicating that with the empirically obtained *U*_*c*_ values, Eq. (5) is able to describe the composition of SDG-generated Au/Ag BNPs. It should be noted that by taking the above mentioned literature value of ${U}_{-}^{Ag}=14 \, {\text{V}}$, we obtain that ${U}_{-}^{Au}\approx 60\, {\text{V}}$, which is reasonably close to the 14–50 V literature range found for the cathode fall voltage of different materials cited above^39–42^. The above results prove that our considerations reflected in Eq. (5) regarding the physical processes of the material erosion in SDGs are able to describe the composition of spark-generated Au/Ag BNPs. This allows for the prediction of the composition of Au/Ag BNPs achievable in SDGs as a function of the generator electrical parameters. As can be seen in Eq. 1, the main parameters are the resistance (*R*), inductance (*L*), and the capacitance (*C*) of the discharge circuit which can affect the current waveform and hence the *k’* factor. These parameters jointly determine whether the spark operates in the underdamped—i.e. oscillatory—or overdamped—i.e. unipolar—regime, therefore setting practical limits to their values in an SDG. From a technical point of view, *R* and *C* are the most conveniently accessible parameters, hence here we only investigate their effect on the Au/Ag composition. We assume an inductance of 1 µH, which is characteristic to our setup and also close to the values reported in the literature^33^. Typically, SDGs are built with—sometimes variable—capacitance in the range of ca. 1–30 nF^28,30,44^, which determines that the transition between the oscillatory and unipolar regimes occurs at a maximum of about 11 Ω. As it was mentioned earlier, the lower limit of the total resistance is defined by the minimum resistance of the circuit and the spark plasma, which in total is usually not much less than 1 Ω. These values set a rational parameter range for an SDG. The theoretically achievable composition values calculated from Eqs. (1) and (5) for the above *R* and *C* range is shown in Fig. 6.

It can be seen that in case of an initially anodic gold electrode and 1 µH inductance, the Ag content of the BNPs can be varied with the control of C and R between about 20% and 100%, the latter indicating the transition to the unipolar spark discharge regime. The 0–20% range is also accessible by simply switching the electrode polarity, i.e. making the silver electrode to be initially anodic. Figure 6 illustrates that Eq. (5) can indeed be used to predict the composition of the Au/Ag BNPs generated at given SDG parameters.

## Conclusions

A gas-phase technique, the spark discharge nanoparticle generation was employed to produce spherical Au/Ag binary nanoparticles (BNPs) with varying composition. The resistance of the discharge circuit was varied in order to change the relative erosion of the Au and Ag electrodes and therefore change the composition of the generated BNPs. The dependence of the average composition of the BNPs on the total resistance and initial electrode polarity was experimentally determined via ICP-MS analysis. It has been shown that the Au content can be continuously varied between 55 and 90 m/m%. By replacing one of the electrodes with an alloy of the two elements, practically the whole 0–100% composition range can be covered. This convenient composition tuning can be achieved without altering the particle size, as proved by the in situ size distribution measurements.

In order to elucidate on the relation of the experimental parameters and the BNP composition, the experimentally acquired data was compared to an existing spark plasma mixing model. This comparison revealed considerable deviations between the measured and calculated values, which led us to reconsider some of the assumptions generally used in the explanation of spark mixing. As a result, a plausible physical explanation was given to the underlying processes, and an extended mixing model was created to predict the Au/Ag BNP composition. This means, that our experimental technique for producing Au/Ag BNPs is also backed up by a validated semiempirical model, which further supports the effective parameterization of experiments aiming to the generation of Au/Ag BNP of virtually any target composition via an environmentally friendly, facile, one-step gas-phase route.

## Material and methods

### Particle generation

**Figure 7:**
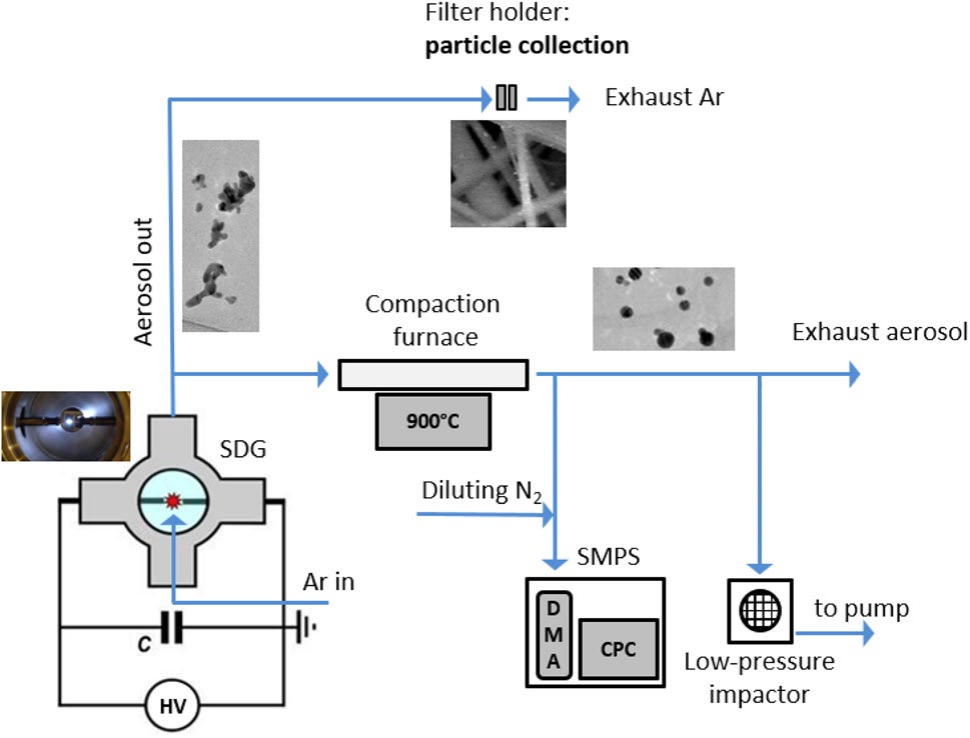
Schematic figure of the particle generation, collection and sampling setup.

The experimental setup was already described elsewhere^28,45^ and schematically shown in Fig. 7. The central part of the spark discharge generator system is a vacuum chamber manufactured by Pfeiffer Vacuum GmbH. It is a KF-sealed, DN-160 sized, cylindrical stainless-steel chamber with four radially oriented KF-40 ports. The chamber was set up in an upright position, with the two large KF-160 ports located on the sides. The applied Ag (99.99% purity, Goodfellow Cambridge Ltd.) and Au (99.99% purity, Kurt J. Lesker Co.) electrode pairs were horizontally positioned and axially aligned. The gap between the two electrodes was 2.0 mm for all experiments and it was controlled by micropositioners (Model K150-BLM-1, MDC Vacuum Ltd.). The diameter of the electrodes with a cylindrical geometry was 3.00 mm.

The argon (99.996% purity, Messer Hungarogáz Ltd.) carrier gas flow was fed in the chamber via the down KF-40 port (upward pointing “crossflow” with injector nozzle), which was employed during NP generation. The gas flow rate was controlled by a mass flow controller (Model GFC16, Aalborg Instruments & Controls, Inc.) and set to 5 standard liter per minute (slm). All experiments were carried out at atmospheric pressure, monitored by a piezo-resistive pressure gauge (Model VD81, Thyracont Vacuum Instruments Gmbh).

The spark discharge was created by a simple capacitor charging circuit. A monolithic, high voltage capacitor (Model 450PM980, General Atomics) with 8 nF capacitance was connected parallel to the spark gap and was charged by a high voltage power supply (Model HCK 800-12500, FuG GmbH). The spark discharge between the electrodes is created when the voltage on the capacitor reaches the breakdown voltage of the gas between the electrodes. The resulting spark discharge is a bipolar, dampened, oscillatory discharge. The repetition rate of the spark discharge (spark repetition rate, SRR) was controlled via the charging current of the capacitor and kept constant at 100 Hz during the experiments, unless stated otherwise. In our measurements the total resistance of the electric circuit was varied by different length of FeCrAl alloy resistance wires (Kanthal, Sandvik Intellectual Property AB, Sweden) in the range of 1–9 Ω. The voltage and current waveforms in the discharge loop were recorded by a 200 MHz digital storage oscilloscope (Model DSOX2024A, Keysight Technologies Inc.) using a broad-band high voltage probe (Model P6015A, Tektronix Inc.) and current probe (Model 110, Pearson Electronics, Inc.), at the same time.

### Particle characterization

The created alloy NPs were collected on a glass microfiber filter (GF/A CAT No. 1820-047, Whatman plc part of GE Healthcare Life Sciences, General Electric) in a filter holder (43303020 Polypropylene Filter Holder, Advantec AS). The sample collection time was 0.50 h and between every sample the plastic tubes and the filter holders were cleaned with a mixture of 50% ethanol (96% purity, Molar Chemical Ltd.) and 50% water solution in an ultrasonic bath (Ultrasonic 300, NEY, now Blackstone-NEY Ultrasonics). The resulting samples were stored in Petri dishes until composition measurements, which were carried out by following the same procedure we have already utilized for SDG-generated Au/Ag NPs^46^. To this end, an inductively coupled plasma mass spectrometer (ICP-MS, 7700x, Agilent Technologies Inc.) was used. Sample dissolution was carried out by aqua regia, prepared freshly from trace quality cc. hydrochloric and cc. nitric acids (VWR Chemicals) under 16 h of contact time. The resulting clear solutions were filtered through 0.22 μm PTFE membrane filters and diluted with trace-quality de-ionized labwater (MilliPore Elix 10 equipped with a Synergy polishing unit, Merck GmbH.) prior to analysis. Multipoint, matrix-matched calibration was performed using certified calibration standards (IV-ICPMS-71A and IV-ICPMS-71C, Inorganic Ventures). ICP-MS plasma and interface parameters were optimized via standard tuning solutions (G1820-60410, Agilent). All measurements were carried out by monitoring the signal of the ^107^Ag and ^197^Au isotopes, in He mode using the ORS^3^ collision cell. Data processing was performed within the Agilent Mass Hunter software. The 99.996% purity argon and 99.999% purity helium gases were purchased from Messer Hungarogáz Kft.

For TEM and EDX analysis, the generated particles were sampled using a lacey carbon copper grid (S166 Lacey Carbon Film 200 Mesh Cu, Agar Scientific Ltd.) by using a low-pressure impactor. The morphology of the nanoparticles was analyzed by high-resolution transmission electron microscopy (TEM, G2 20 X-TWIN HR-TEM, FEI Tecnai, Thermo Fisher Scientific). The energy dispersive X-ray (EDX, Quantax XFlash 6T, Bruker Corporation) profiles were obtained at an accelerating voltage of 200 kV.

The size distribution of the spark generated particles was measured using a scanning mobility particle sizer (SMPS) system consisting of an electrostatic classifier (3082, TSI, USA), an ultrafine condensation particle counter (CPC, 3756, TSI, USA), and an aerosol charge neutralizer (Kr-85, NRD, USA). The SMPS system was operated with a sample flow of 0.3 L/min, a sheath flow of 3 L/min, and a scan time of 60 s (measurement range: 4.61–151.20 nm).

Prior to size distribution measurement and particle sampling for morphology characterization the generated aerosol passed through a compaction furnace (EHA 12/300B, Carbolite Gero GmbH.) equipped with a ceramic tube set to 900 °C in order to compact the produced nanoaggregates. It has been confirmed via ICP-MS analysis that the compaction did not change the average composition of the generated particles collected on a filter.

## Supplementary Information

Supplementary Information.
