# Supplementary material for: Full range tuning of the composition of Au/Ag binary nanoparticles by spark discharge generation
Source: Sci Rep. 2021 Mar 4;11:5117. doi: 10.1038/s41598-021-84392-6 (PMC7970845; doi:10.1038/s41598-021-84392-6)
Supplement: Supplementary file 1 — Supplementary Information. [file 41598_2021_84392_MOESM1_ESM.docx]

**Supplementary Information**

Full range tuning of the composition of Au/Ag binary nanoparticles by spark discharge generation

Attila Kohut^1,2^, Lajos Péter Villy ^1^, Albert Kéri^2,3^, Ádám Bélteki^3^, Dániel Megyeri^1^, Béla Hopp^1,2^, Gábor Galbács^1,2*^, and Zsolt Geretovszky^1,2*^

^1^Department of Optics and Quantum Electronics, University of Szeged, Dóm sq. 9, 6720 Szeged, Hungary

^2^Department of Materials Science, Interdisciplinary Excellence Centre, University of Szeged, 6720 Szeged, Dugonics sq. 13, Hungary

^3^Department of Inorganic and Analytical Chemistry, University of Szeged, Dóm sq. 7, 6720 Szeged, Hungary

*corresponding author, email: (G. Zs.) [gero@physx.u-szeged.hu](mailto:gero@physx.u-szeged.hu), (G. G.) [galbx@chem.u-szeged.hu](mailto:galbx@chem.u-szeged.hu)

**Size distribution of the generated nanoparticles**


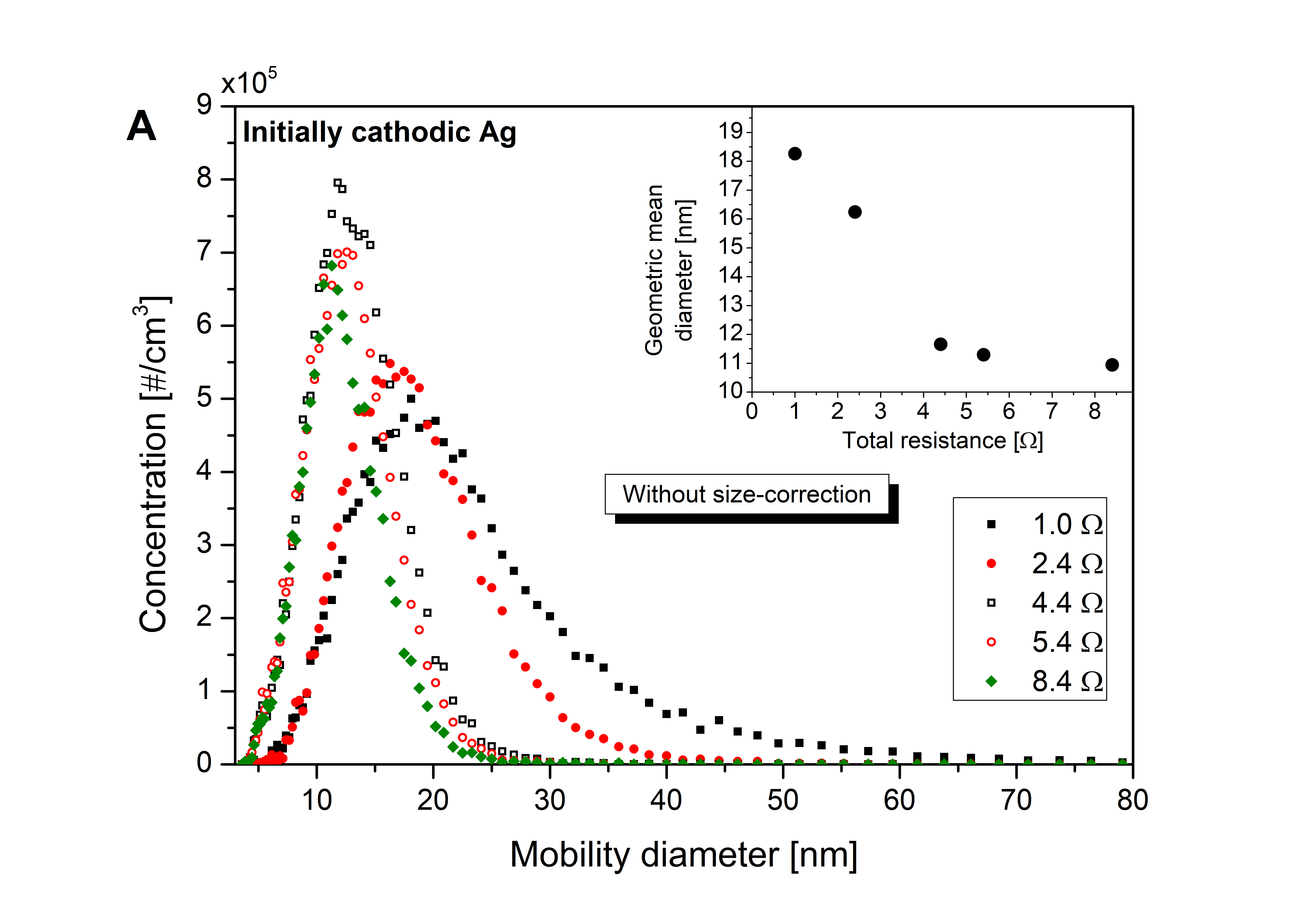

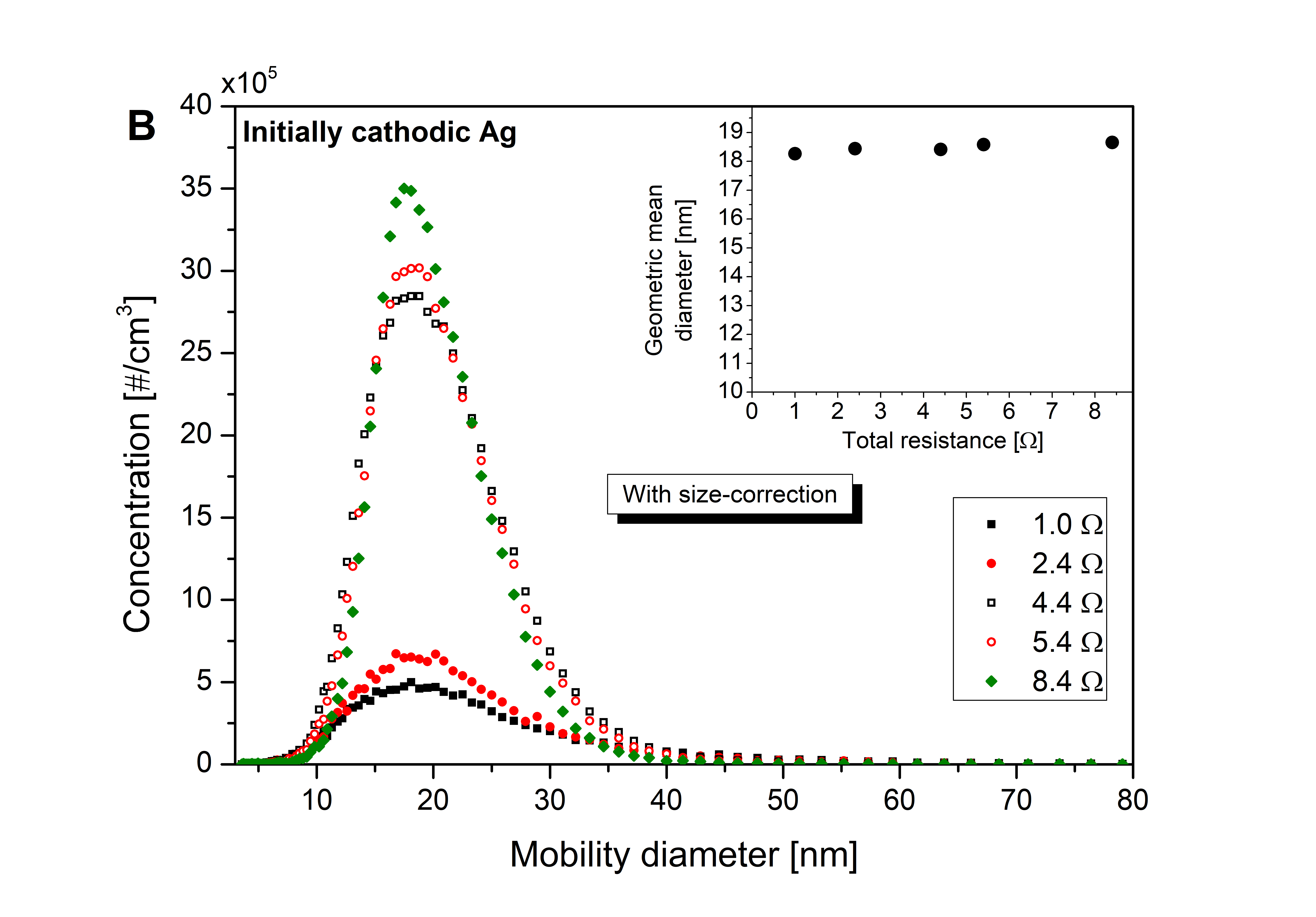


Figure S1. Size distribution of the Au/Ag BNPs generated at varying resistance at fixed generator parameters (A) and with varying generator parameters to keep the particle size constant (B). Variation of the geometric mean particle diameter is shown
 in the insets in both graphs.

**Ratio of particle mass obtained with different initial electrode polarities**


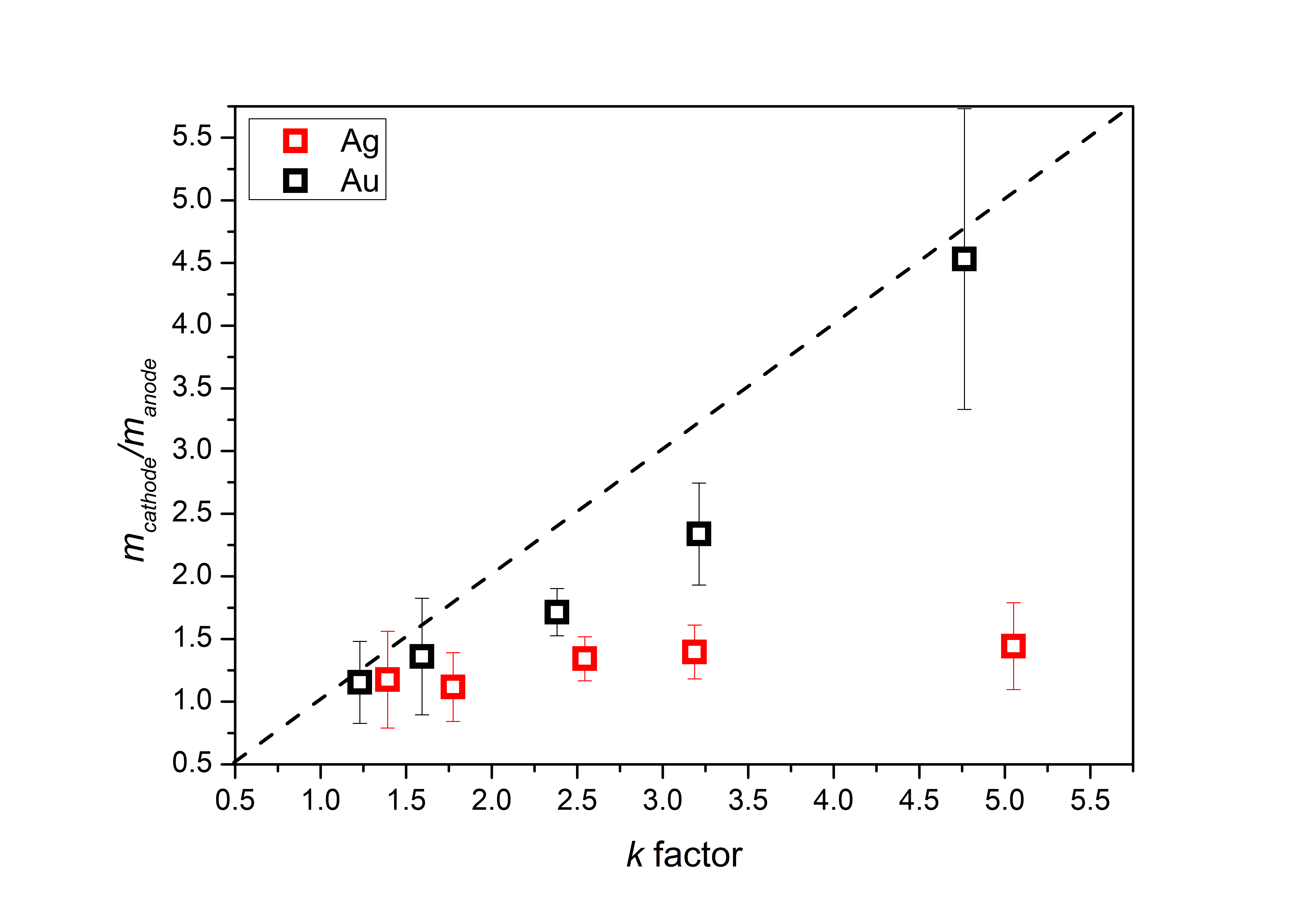


Figure S2. Ratio of particle mass eroded when the electrode is initially cathodic and anodic, respectively, as a function of the *k* factor for Au and Ag electrode materials.

**General considerations regarding the spark mixing model**

Due to the oscillatory nature of the spark the initial polarity of the electrodes – which will be noted as subscript *C* and *A* for the cathode and anode, respectively – changes over time. Therefore, we distinguish between the energy delivered to the initially cathodic electrode in its momentary cathodic and anodic periods as:

$E_{-}^{C}=U_{-}^{C}\cdot\int I_{-}(t)dt$ and $E_{+}^{C}=U_{+}^{C}\cdot\int I_{+}(t)dt$, (S1)

where $U_{-}^{C}$ and $U_{+}^{C}$ are the typical cathode and anode voltage drop for the electrode material having the initially cathodic polarity, respectively; and $\int I_{-}(t)dt$ and $\int I_{+}(t)dt$ are the integral of the current corresponding to the initial and the reversed polarity, respectively. We assume that the voltage drop is constant in the current range covered here. The energy delivered to the initially anodic electrode in its momentary cathodic and anodic periods is the following:

$E_{-}^{A}=U_{-}^{A}\cdot\int I_{+}(t)dt$ and $E_{+}^{A}=U_{+}^{A}\cdot\int I_{-}(t)dt$, (S2)

where $U_{-}^{A}$ and $U_{+}^{A}$ are the typical cathode and anode voltage drop for the electrode material having the initially anodic polarity, respectively.

We assume that the overall erosion efficiency of a given generator setup is constant and denoted by *α*. We also assume that the erodibility of the electrodes – i.e. the mass eroded by unit spark energy – can be described by a material constant *C*, which is practically equals to the reciprocal of the heat of vaporization of a given electrode material. As a result, the mass eroded from the initially cathodic electrode can be written as:

$m^{C}=C^{C}\cdot\alpha\cdot\left( U_{-}^{C}\cdot\int I_{-}\left( t \right)dt+U_{+}^{C}\cdot\int I_{+}(t)dt \right)$ (S3)

while the mass eroded from the initially anodic electrode is:

$m^{A}=C^{A}\cdot\alpha\cdot\left( U_{-}^{A}\cdot\int I_{+}(t)dt+U_{+}^{A}\cdot\int I_{-}(t)dt \right)$ (S4)

**Derivation of Eq. 4 in the main text**

First, let’s introduce the ratio of the mass eroded from the initial cathode and anode during the total duration of sparking as:

$m^{C/A}=\frac{m^{C}}{m^{A}}$ (S5)

and the ratio of the integral of the current corresponding to the two polarities as:

$k^{'}=\frac{\int I_{-}(t)dt}{\int I_{+}(t)dt}$ (S6)

If we consider the case when identical electrodes are used, e.g. $U_{-}^{C}=U_{-}^{A}\equiv U_{-}$ and
 $U_{+}^{C}=U_{+}^{A}\equiv U_{+}$, by dividing Eq. S3 by Eq. S4 and substituting Eqs. S5, S6 into the equation one can derive:

$\frac{U_{-}}{U_{+}}=\frac{m^{C/A}\cdot k^{'}-1}{k'-m^{C/A}}$, (S7)

which gives the ratio of the cathodic and anodic voltage drops for a given electrode material.

**Derivation of Eq. 5 in the main text**

Let’s introduce the reciprocal of Eq. S7 for both electrodes as:

$U_{+/-}^{C}=\frac{U_{-}^{C}}{U_{+}^{C}}$ and $U_{+/-}^{A}=\frac{U_{-}^{A}}{U_{+}^{A}}$ (S8)

By dividing Eq. S3 and Eq. S4 and substituting Eqs. S6 and S8 one can derive that

$\frac{m^{C}}{m^{A}}=\frac{C^{C}}{C^{A}}\cdot\frac{U_{-}^{C}}{U_{-}^{A}}\cdot\frac{k^{'}+U_{+/-}^{C}}{U_{+/-}^{A}\cdot k^{'}+1}$ (S9)

If we denote the relative erosion of the initially cathodic electrode with respect to the total erosion of the two electrodes as (all quantities meant for the whole duration of sparking):

$\varphi_{C}=\frac{m^{C}}{m^{C}+m^{A}}$, (S10)

then rearranging Eq. S9 leads to:

$\varphi_{C}=\frac{1}{\frac{1}{\frac{C^{C}}{C^{A}}\cdot\frac{U_{-}^{C}}{U_{-}^{A}}\cdot\frac{k^{'}+U_{+/-}^{C}}{U_{+/-}^{A}\cdot k^{'}+1}}+1}$ (S11)
